# Review of factors affecting virus inactivation in aerosols and droplets

**DOI:** 10.1098/rsif.2024.0018

**Published:** 2024-06-26

**Authors:** Alexandra K. Longest, Nicole C. Rockey, Seema S. Lakdawala, Linsey C. Marr

**Affiliations:** ^1^ Department of Civil and Environmental Engineering, Virginia Tech, Blacksburg, VA, USA; ^2^ Department of Civil and Environmental Engineering, Duke University, Durham, NC, USA; ^3^ Department of Microbiology and Immunology, Emory University, Atlanta, GA, USA

**Keywords:** virus, inactivation, humidity, aerosol, droplet

## Abstract

The inactivation of viruses in aerosol particles (aerosols) and droplets depends on many factors, but the precise mechanisms of inactivation are not known. The system involves complex physical and biochemical interactions. We reviewed the literature to establish current knowledge about these mechanisms and identify knowledge gaps. We identified 168 relevant papers and grouped results by the following factors: virus type and structure, aerosol or droplet size, temperature, relative humidity (RH) and evaporation, chemical composition of the aerosol or droplet, pH and atmospheric composition. These factors influence the dynamic microenvironment surrounding a virion and thus may affect its inactivation. Results indicate that viruses experience biphasic decay as the carrier aerosols or droplets undergo evaporation and equilibrate with the surrounding air, and their final physical state (liquid, semi-solid or solid) depends on RH. Virus stability, RH and temperature are interrelated, but the effects of RH are multifaceted and still not completely understood. Studies on the impact of pH and atmospheric composition on virus stability have raised new questions that require further exploration. The frequent practice of studying virus inactivation in large droplets and culture media may limit our understanding of inactivation mechanisms that are relevant for transmission, so we encourage the use of particles of physiologically relevant size and composition in future research.

## 1. Introduction

Airborne transmission of pathogens is a major concern for public health because they can spread quickly and easily from person to person, even without close physical contact. Viruses that spread through the air have been responsible for global pandemics, such as the coronavirus disease 2019 (COVID-19) pandemic caused by severe acute respiratory syndrome coronavirus 2 (SARS-CoV-2) and the Great Flu of 1918 pandemic caused by influenza virus, and seasonal epidemics.

While multiple types of evidence show that viruses can spread through the air [1–5], some mechanistic details of transmission are not fully understood. The airborne route of transmission begins with the shedding of the virus in respiratory aerosol particles and droplets. During their transport through the environment, they undergo physical and chemical transformations that may lead to virus inactivation [1,6–8]. The mechanisms driving the inactivation of viruses in aerosols and droplets are still unknown. Various factors, including humidity, temperature, droplet pH and salinity, have been shown to affect the stability of viruses in the air or on surfaces [9–11].

Studies of the stability of aerosolized viruses were common in the 1960s and 1970s, and often employed a rotating drum, sometimes called a ‘Goldberg drum’ after the first researcher who described it [12–17]. Perhaps owing to a shift in attention by the medical and research communities towards chronic diseases in subsequent decades, interest in airborne pathogen transmission languished for several decades afterwards. The COVID-19 pandemic renewed attention to this topic. Given the resurgence of interest, we critically reviewed the literature to synthesize older and more recent results and to identify knowledge gaps on virus inactivation in aerosols and droplets. We organized the discussion into the following categories: virus type and structure, aerosol or droplet size, temperature, relative humidity (RH) and evaporation, chemical composition of the aerosol or droplet, pH and atmospheric composition. Many of these factors are intricately intertwined, so some overlap in topics is unavoidable. We conclude with a list of recommendations to apply this knowledge effectively and to advance our understanding of the mechanisms of virus inactivation.

## 2. Search strategy and data extraction

The three databases used for this review include Web of Science (WOS), PubMed and SCOPUS. Each database was searched on 5 December 2022. Electronic supplementary material, figure S1 summarizes the approach and criteria applied when selecting articles for inclusion in the critical review, and electronic supplementary material, table S1 summarizes the search phrases for each database. Additional details of our review can be found in the electronic supplementary material. We grouped papers by the identified factors affecting virus inactivation in aerosols and droplets (table 1).

**Table 1.** Factors and their effects on virus inactivation.

factor	description
virus type and structure	effect of different virus structures (e.g. enveloped, non-enveloped) or different virus families, genera, or strains
aerosol or droplet size	effect of initial particle size; changes in size over time
temperature, RH and evaporation	effect of temperature and/or RH over time; effect of evaporation and/or drying over time; physical state of aerosols and droplets based on RH and evaporation
chemical composition of the aerosol or droplet	effect of chemical composition of suspending medium and additives
pH	effect of acidification or alkalization; speculation about pH change
atmospheric composition	effect of gases or particles in the surrounding air

## 3. Virus type and structure

The majority of existing studies on the environmental stability of viruses in aerosols and droplets focus on influenza virus and SARS-CoV-2. Other viruses have been investigated to a lesser extent, and in some cases, surrogates are used to mitigate biosafety risks in the laboratory. Owing to the strong influence of RH observed in many studies, we have separated the discussion of some results into categories of low (less than or equal to 40%), medium (40–75%) and high RH (greater than or equal to 75%).

### 3.1. Enveloped versus non-enveloped

The macroscale structure of viruses, namely the presence or absence of an envelope, may affect their stability. Non-enveloped viruses contain the viral genome enclosed in a protein capsid, while enveloped viruses are further surrounded by a membrane that contains lipids and proteins. The envelope is thought to improve host interaction but makes the virus more vulnerable to environmental factors including extreme pH, heat, dryness and disinfectants compared with non-enveloped viruses. In general, enveloped viruses appear to be more sensitive to changes in pH and to display different trends in inactivation with RH compared with non-enveloped viruses [18–20], although disparities in RH-dependent decay exist even among viruses that share either an enveloped or non-enveloped structure [13,21–29]. These differences in decay extend beyond RH and are evident when adjusting salt concentrations [9,30–33]. The effects of temperature, protein and surfactant levels, pH, aerosolization method, particulate matter and ozone inactivation on virus inactivation have also been considered [9,30,34–41]. The most direct comparisons of enveloped and non-enveloped viruses have involved the bacteriophages Phi6 and MS2. Network diagrams, separated into low, medium and high RH, illustrate differences in these two viruses’ response to changes in temperature, particle chemistry, atmospheric composition and particle size (electronic supplementary material, figure S2). For human viruses, few studies have directly compared the inactivation of non-enveloped and enveloped viruses.

### 3.2. Influenza virus

Influenza A virus (IAV) and influenza B virus (IBV) can cause seasonal flu epidemics in humans, but IAV is the only influenza virus type known to cause pandemics [42]. IAVs are divided into subtypes based on the two surface proteins of the virus: haemagglutinin (HA), of which there are 19 different subtypes, and neuraminidase (NA), of which there are 11 different subtypes [42]. Seasonal and pandemic human IAVs include the following subtypes: H1N1, H3N2 and H2N2. HA subtypes 1–16 circulate in avian species including migratory birds, and subtypes 17 and 18 have been found in bats [42].

The environmental stability of influenza viruses differs not only by host origin but also by virus subtype [43–46], as described in greater detail in subsequent sections. Influenza viruses can be explored further within virus subtypes by comparing laboratory-adapted strains isolated in 1933 and 1934 (e.g. W.S./WSN and PR8) and contemporary human seasonal IAV strains. Most studies of inactivation have focused on lab-adapted H1N1, mainly at low and medium RH. However, lab-adapted strains are not representative of the seasonal IAVs as the former have been extensively passaged in eggs and mouse models and thus no longer infect humans or human respiratory cells with a similar capacity as do isolated seasonal influenza viruses [47,48]. Thus, we recommend limiting the use of lab-adapted strains in investigating environmental persistence.

**Figure 1.**
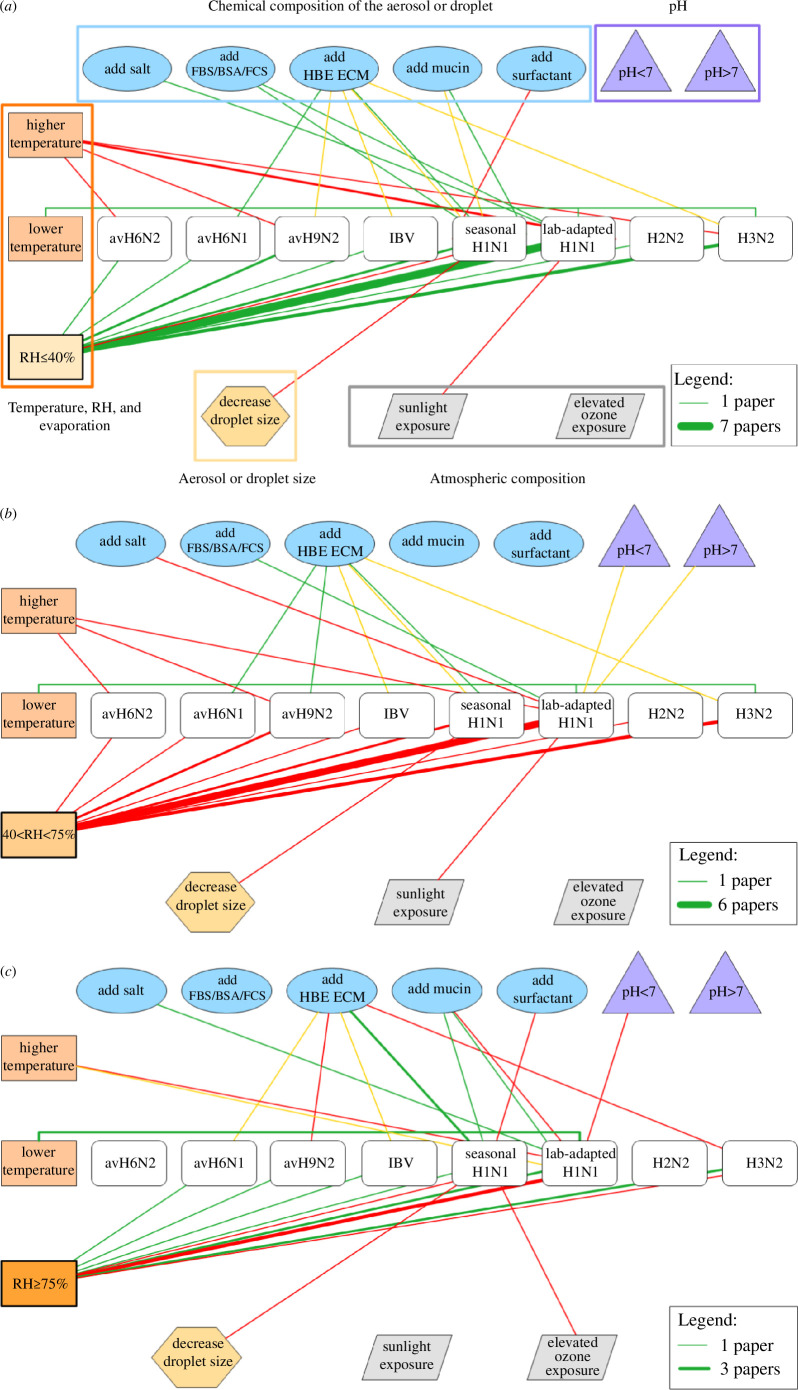
Network connection diagrams for influenza viruses. Factors impacting influenza virus stability at (*a*) low, (*b*) medium and (*c*) high RHs. Higher and lower temperatures refer to temperatures above and below the room temperature range (20–24°C), respectively. Green, red and yellow lines represent protective, harmful and neutral effects on virus viability, respectively. The thickness of the lines corresponds to the number of papers reporting a particular effect. The effects are defined by statistically significant differences in results or apparent trends when statistics are not available. Calculated exponential decay constants for each line are provided in electronic supplementary material, table S4. Virus subtypes and strains: avian influenza virus H6N2 (avH6N2)[A/Turkey/Mass/3740/65 (H6N2)] [44], avian influenza virus H6N1 (avH6N1)[A/Shorebird/DE Bay/230/2009 (H6N1)] [43], avian influenza virus H9N2 (avH9N2)[A/Chicken/Henan/98 (H9N2), A/Shorebird/DE/127/2003 (H9N2)] [43,44], influenza B virus (IBV)[B/Texas/02/2013] [43], human seasonal influenza A virus H1N1 (seasonal H1N1)[A/California/07/2009 (H1N1), A/Michigan/45/2015 (H1N1), A/Mexico/4108/2009 (H1N1)] [21,34,43,49–51], lab-adapted IAV H1N1 strains (lab-adapted H1N1)[A/PR/8/34 (H1N1), A/WS/33 (H1N1)] [13,22,30,31,52–56], human seasonal influenza A virus H2N2 (H2N2)[A2/Japan/305/1957] [57], and human seasonal influenza A virus H3N2 (H3N2)[A/Perth/19/09 (H3N2), A/Udorn/307/1972 (H3N2), A/Panama/2007/99 (H3N2)] [35,43,58]. Figure created using ConceptDraw DIAGRAM, by CS Odessa.

Figure 1 is a network diagram that shows the effects of different factors on influenza virus stability across low, medium and high RH. Different virus types span the centre of the figure, and different physical and chemical factors surround them. The lines connecting a virus and a factor indicate whether the factor is protective (green), harmful (red) or neutral (yellow), and the thickness of the line represents the number of studies that report a particular relationship. In detail, these figures depict the types and frequency of factors tested as well as how the effects of these factors can vary across humidity levels.

The factors listed in table 1 are highlighted in figure 1*a*. These also correspond to the section headings in this paper. Humidity, highlighted along with temperature by an orange box, has been studied the most, with a general trend of being protective at low RH (figure 1*a*: mostly green lines to different virus types), harmful at medium RH (figure 1*b*: mostly red lines to different virus types) and having variable effects at high RH (figure 1*c*: a mix of green and red lines to different virus types). The effect of salt, inside the blue box labelled ‘Chemical composition of the aerosol or droplet’, varies across RH; at low and high RH, it is protective, whereas at medium RH, it is harmful to lab-adapted H1N1. Human bronchial epithelial extracellular matrix (HBE ECM), inside the blue box labelled ‘Chemical composition of the aerosol or droplet’, is protective or neutral to avH9N2 and H3N2 but harmful at high RH. These relationships and others are explored in greater detail in subsequent sections.

The network diagrams illustrate the complexity of interactions among different factors and the major role of humidity, which we posit in §5 is related to its effect on the physical state of the particle. The diagrams also reveal that relationships can differ by influenza virus subtype and general conclusions cannot be made across the family.

### 3.3. Coronaviruses

**Figure 2.**
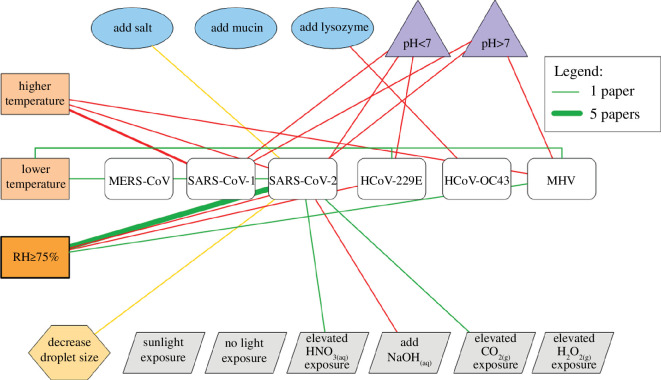
Network connection diagram for coronaviruses. Factors impacting coronavirus stability at high RH. Higher and lower temperatures refer to temperatures above and below the room temperature range (20–24°C), respectively. Green, red and yellow lines represent protective, harmful and neutral effects on virus viability, respectively. The thickness of the lines corresponds to the number of papers reporting a particular effect. The effects are defined by statistically significant differences in results or apparent trends when statistics are not available. Calculated exponential decay constants for each line are provided in electronic supplementary material, table S5. Variants of SARS-CoV-2 in the studies include WA1/2020, REMRQ0001, Delta, England-2, O.S. and BetaCoV-2020 [10,21,51,55,61–64,67–80]. Additional network diagrams at low and medium RHs are available in electronic supplementary material, figure S3. Figure created using ConceptDraw DIAGRAM, by CS Odessa.

SARS-CoV-2 is a member of the *Coronaviridae* (CoV) family, which includes severe acute respiratory syndrome coronavirus 1 (SARS-CoV-1) and Middle East respiratory syndrome coronavirus (MERS-CoV), both of which have caused large outbreaks [59]. In addition to pandemic and emerging CoV threats, human (HCoV) 229E, OC43, NL63 and HKU1 circulate in the human population each year [60]; however, the environmental stability of these viruses has not been studied as extensively compared with SARS-CoV-2 [55,61–64]. Since emerging in 2019, SARS-CoV-2 has evolved, and newer variants, such as Alpha, Beta and Delta, have been shown to have similar airborne stability at low and high RH [10,65,66]. Differences in stability between variants increase when other environmental factors (electronic supplementary material, table S5), like simulated sunlight and exposure to alkaline conditions, are considered, but the general trends are the same across different variants (figure 2 and electronic supplementary material, figure S3) [65,67]. The effects of pH and atmospheric conditions have almost exclusively been studied at high RH, probably because the liquid state of the particles, discussed in §5, facilitates such experiments.

### 3.4. Other viruses

Our literature search identified several other viruses that have been the subject of at least four research papers each. These include rhinovirus, respiratory syncytial virus (RSV), bacteriophages and rotavirus. A full list of viruses and the number of studies that investigated them is in electronic supplementary material, table S6.

Viruses and other pathogens are categorized into biosafety levels (BSL 1–4) based on infectivity, severity of disease and other risk factors that can be simplified as the degree of hazard they present. Researchers often try to limit their work with BSL-3 and BSL-4 pathogens, such as SARS-CoV-2 and Ebola virus, respectively, and use surrogates at lower biosafety levels instead. Bacteriophages, or viruses that infect bacteria, are frequently used as surrogates because they are not pathogenic to humans and require lower levels of biocontainment [9,23,81]. Phi6 and MS2 are commonly used, although few studies have directly compared them against pathogenic viruses of interest. Comparing the inactivation of SARS-CoV-2, a seasonal influenza virus and Phi6, French *et al*. [21] found that influenza virus was a superior surrogate for SARS-CoV-2 compared with Phi6. In a related study, researchers compared the decay of mouse hepatitis virus (MHV) and SARS-CoV-2 and suggested that the use of closely related viruses within the same family may more accurately represent the phenotypes of emerging viral threats compared with bacteriophage surrogates [75]. Surrogates need to be chosen carefully and their results interpreted with caution since macroscale and molecular-scale features, such as genomes and proteins, of the virus may influence environmental stability.

## 4. Aerosol or droplet size

### 4.1. Aerosol and droplet size distribution

Traditionally, aerosol and droplet transmission were distinguished by particle size and distance travelled. Here, we define ‘aerosol particles’, or ‘aerosols’ for short, as having a diameter less than 100 μm and ‘large droplets’ as having a diameter greater than or equal to 100 μm [82,83]. We use the word ‘particle’ to refer to both these objects, regardless of size and the word ‘droplet’ to refer to everything that is wet, regardless of size. When referring to the complete physical structure of a virus in a respiratory particle, we use the term ‘virion.’ Table 2 shows the physical properties of example aerosols and large droplets, as well as pipetted droplets that are used in laboratory experiments. While the initial diameters of the droplets span more than three orders of magnitude, the surface areas and volumes span 6–11 orders of magnitude. The enormous range of values has implications for virus inactivation through its effect on evaporation rates and mass transfer across the air–particle interface.

**Table 2.** Physical properties of model respiratory particles over a range of initial diameters, including their volume (V), surface area (SA) and surface area to volume ratio (SA/V) before and after evaporation to a dry state.

object^a^	initial diameter (µm)	initial V (µl)	initial SA^b^ (µm^2^)	initial SA/V (µm^−1^)	dry diameter^c^ (µm)	dry V (µl)	dry SA (µm^2^)	dry SA/V (µm^−1^)
aerosol	1	5.2 × 10^−10^	3.14	6.0	0.2–0.4	4.2×10^−12^−3.4×10^−11^	0.13–0.50	30–15
aerosol	50	6.5 × 10^−5^	7.9 × 10^3^	1.2 × 10^−1^	10–20	5.2×10^−7^–4.2×10^−6^	3.1×10^2^−1.3×10^3^	0.6–0.3
large droplet	100	5.2 × 10^−4^	3.1 x 10^4^	6.0 × 10^−2^	20–40	4.2×10^−6^–3.4×10^−5^	1.3×10^3^−5.0×10^3^	0.3–0.15
large droplet	500	6.5 × 10^−2^	7.9 x 10^5^	1.2 × 10^−2^	100–200	5.2×10^−4^−4.2×10^−3^	3.1×10^4^−1.3×10^5^	0.06–0.03
pipetted droplet	1563	1	3.8 × 10^6^	3.8 × 10^−3^	313–625	0.008–0.064	1.5×10^5^−6.1×10^5^	0.019–0.010
pipetted droplet	5759	50	5.2 × 10^7^	1.0 × 10^−3^	1152–2304	0.4–3.2	2.1×10^6^−8.3×10^6^	0.005–0.003

^a^ Assuming spherical geometry for the aerosols and large droplets and hemispherical geometry for the pipetted droplets deposited on a flat surface.

^b^ Exposed (curved) surface area for the pipetted droplet.

^c^ Assuming a ratio of dry diameter to initial diameter for respiratory droplets (spherical and hemispherical) of 0.2–0.4 from Merhi *et al*. [84] and Marr *et al*. [11].

Respiratory droplets spanning a wide range of sizes are released into the air during breathing, talking, singing, coughing, sneezing and other activities [85]. The droplets shrink from their initial size as water evaporates until they reach equilibrium with the surrounding air [11]. Many factors dictate the initial and final equilibrium sizes of respiratory droplets, such as the site of origin, chemical composition of the fluid and RH [7,8,84,86,87], and all of these can affect the fate of a virion carried in the droplet. The three principal zones of droplet generation are the bronchiolar region and the laryngeal region, which both produce smaller aerosols (modal droplet diameter of 1–2 μm), and the combined oral cavity and nasal region that produce large droplets (modal droplet diameter greater than 100 μm) [86,88]. The salt composition depends on the site of origin of the droplets, with large droplets of saliva dominated by potassium (ratio approx. 3:1 of K^+^:Na^+^) and small droplets from the nasal cavity dominated by sodium (ratio approx. 4:1 of Na^+^:K^+^) [84,86,87]. The salt composition affects the efflorescence RH, which impacts the relationship between RH and the equilibrium size of the droplet [86].

While human viruses, ranging in size from 0.02 to 0.2 μm, can be much smaller than the particles that contain them, there is limited information about the size distribution of particles that actually carry virions [56]. Furthermore, researchers have not yet been able to measure the number of virions present in an individual particle [89–91]. More research is needed to understand the ‘payload’ of virions within particles as well as what size particles contribute most to transmission.

### 4.2. Particle size and virus inactivation

Virus stability has been shown to vary with carrier particle size. Ud-Dean [92] hypothesized that initial droplet size is important because the fate of a virion is determined by the ratio of the time for water to diffuse into the envelope to the time for the droplet to diminish to the size of the virion along with a thin molecular film around it. However, the model considered inactivation owing only to the actions of water molecules and did not account for other components in the droplet. Nonetheless, time scales for diffusion are probably important for virus inactivation.

Virus stability in aerosols of different sizes has been investigated in a few studies. While some studies observed size-dependent decay in aerosols [84,93,94], Alexander *et al.* [75] reported a similar loss of infectivity of MHV in 51 and 62 μm particles; this range of diameters is very small compared with that of all respiratory particles. Seemingly conflicting results between studies examining particle size and virus stability could be owing to different aerosolization and collection techniques or the range of particle sizes examined.

Among the few studies that have compared virus decay in both aerosols and large droplets, results vary. Trends in RH-dependent decay of Phi6 and influenza virus were similar in aerosols and droplets [36,49]. However, other researchers found higher stability of viruses in aerosols than in large droplets across RHs from 30% to 90%. One study that compared MHV stability in deposited aerosols and 50 μl droplets found similar infectivity on plastic but increased stability in the aerosols compared with large droplets on copper [79]. These and additional studies on virus stability in aerosols and droplets were performed with the virus in cell culture media [51,62,95], and whether the results can be extended to physiological fluids is not known. Generally, the kinetics of virus decay depend on the droplet size (figures 1*a–c* and 2, electronic supplementary material, figures S2 and S3) [21,84], though this is not a dominant factor affecting inactivation.

The drivers of inactivation appear to be similar regardless of particle size; however, the surface-area-to-volume ratio will affect inactivation kinetics, particularly those involving mass transfer across the air–particle interface or phenomena at the interface. With this in mind, researchers should move towards techniques that produce smaller, more physiologically relevant particles when analysing viral persistence in the environment. Additional comparisons between virus inactivation in aerosols versus pipetted droplets are also needed to determine how results involving the latter, which are far easier to handle, can be translated to the former.

### 4.3. Experimental limitations for particles of physiologically relevant size

One reason for uncertainty about the relationship between particle size and virus inactivation is the difficulty in replicating realistic respiratory droplets experimentally because of their complex chemical composition. Studies have typically used one of two methods to produce particles: nebulizers to produce aerosols or pipettes to create droplets of volumes ranging from 1 to 50 µl. Nebulizers produce a polydisperse aerosol whose size distribution is affected by the suspension media [96]. Microdroplet dispensers enable the generation of individual droplets of controlled size. A limitation of studying a small population of droplets is that the absolute number of infectious virions in a sample could be low, limiting detection of decay beyond about one order of magnitude. For example, if a sample contained 50 plaque-forming units (PFU) initially, then only 1.4 log_10_ decay could be measured before the infectious virus concentrations in the sample would drop below the limit of detection of a standard plaque assay. In a sample with 5000 PFU initially, up to 3.4 log_10_ decay could be detected before reaching the limit of detection. Other limits include dispensers that are prone to clogging and momentary exposure of virions to complex fluid dynamics with effects that are hard to predict.

### 4.4. Summary of particle size effects on inactivation

While particle size does not directly influence virus stability in the environment, size affects the rate of physical and chemical changes of respiratory particles. Large droplets are useful in experimental studies as models to investigate the interactions occurring within particles. However, their microscale physical and chemical properties may differ from those of smaller particles that are more relevant for transmission. Conclusions about viral persistence in large droplets may not apply to smaller particles, so such results should be interpreted with caution. The size of particles that contribute most to transmission remains unknown and could vary between viruses, depending on environmental and host factors, including the anatomical site of infection. Studies with large droplets should be extended to particles of physiologically relevant size to advance our understanding of transmission.

## 5. Temperature, RH and evaporation

It is well established that temperature and RH both impact virus viability. Some studies have found that temperature has a greater effect on survival than does humidity, but because most transmission occurs indoors where the range in temperature is relatively small, RH potentially has a more relevant influence on virus stability [11,76,97,98]. The relationships between temperature, RH and virus stability have been extensively studied, although a mechanistic understanding remains elusive.

**Figure 3.**
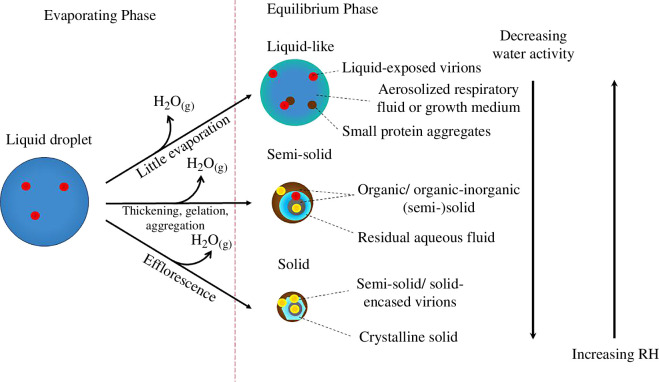
Biphasic decay of viruses, and the final physical states of particles in the equilibrium phase. The final physical state of particles depends on ambient RH. At high RH, the liquid-like state subjects the virions to reactions with solutes, but their concentrations are low. As RH decreases, the organic and organic–inorganic compounds can form an amorphous, (semi-) solid state that exhibits solid-like properties, which can protect the virions by hindering the diffusion of reactants. At low RH, enough water is lost that the crystalline, formed from efflorescence, or amorphous solid will inhibit inactivation. This figure is adapted from Huynh et al. [100], used under CC BY 4.0 [100].

When expelled from the respiratory tract, aerosols and droplets are initially in the liquid state, as they are composed of respiratory fluid and/or saliva, perhaps enriched in certain components and depleted in others during the aerosolization process. As the particles transition from a RH of approximately 100% and temperature of 37°C in the respiratory tract to lower RH and temperature (20–24°C) in ambient air, water evaporates until the vapour pressure at the particle surface reaches equilibrium with that of the surroundings [99]. Depending on ambient RH, the particles’ final state can be liquid, semi-solid or solid [30,58,100–102]. These states correspond to high, medium and low RH, respectively (figure 3) [100]. The effect of physical state on virus viability is discussed in §5.2.

### 5.1. Temperature and virus inactivation

Across virus types and strains, increasing temperature (range of 27–70°C) leads to an increase in viral decay owing to higher rates of protein and nucleic acid denaturation [6,11,14,28,41,44,51,64,68–71,76,84,94,97,103,104]. Numerous studies have observed that viruses are more stable at lower temperature (range of 4–19°C) [13,31,35,36,40,61,69,71,105–109].

Temperature can also affect the drying morphology of a deposited droplet. The area of the crystalline zone was found to shrink with increasing temperature, and additionally, the morphology of the crystalline structures changed from dendritic at 20°C to cell-like at 25–40°C [110]. The difference may be owing to convective effects at higher temperatures, causing the formation of instabilities and convective cells in the drying droplet [110]. How this phenomenon affects the virus within droplets is unknown, but it could impact the location of the virus within a dried droplet, which could in turn affect the stability of the virus and whether it is protected from environmental drivers of decay.

### 5.2. RH, the physical state of particle and virus inactivation

RH, the physical state of the particle and virus stability are intricately intertwined. Huynh et al. [100] suggested that there are four equilibrium RH regions that dictate virus viability: (i) physiological conditions (99–100% RH) with solute concentrations that are harmless to the virus, (ii) concentrated conditions (approx. 75% RH) with high solute concentrations in a liquid-like state, (iii) semi-solid conditions (approx. 45–75% RH) with increasing viscosity and decreasing diffusion offsetting the effect of increasing solute concentrations, and (iv) dry conditions (less than 45% RH) with the formation of crystalline or amorphous solids that inhibit inactivation. The first two regions are combined in the ‘liquid-like’ physical state of the particle in figure 3. These regions may not be relevant for all viruses, however, as researchers have observed different relationships between virus viability and RH depending on the type of virus.

Four different types of relationships between RH and virus viability have been observed: (i) monotonically decreasing viability (increasing inactivation) with increasing RH, (ii) monotonically increasing viability (decreasing inactivation) with increasing RH, (iii) ‘U-shaped’ virus viability (i.e. elevated viability at low and high RHs, with decreased viability at medium RH), and (iv) no difference in viability as a function of RH. Because measurements of infectivity are usually collected after many minutes to hours in experiments, these results usually incorporate multiple phases of decay (§5.3), although one older study and some recent studies include sufficient temporal resolution to distinguish biphasic or triphasic decay [10,13,21,71,74]. Typically, researchers have observed increasing infectivity with increasing RH in non-enveloped viruses, for example poliovirus [13,24,52], foot-and-mouth disease virus (FMDV) [111,112] and rhinovirus [102,113], and a U-shaped trend in infectivity among enveloped viruses, for example influenza virus [30,43,114], vesicular stomatitis virus (VSV) [77,115] and SARS-CoV-2 [6,71] (figures 1*a–c* and 2, electronic supplementary material, figures S2a–c and S3a,b). Some studies reported decreasing infectivity with increasing RH for enveloped viruses, such as influenza virus and Phi6, but these studies employed a maximum RH of 70–80% and therefore missed the behaviour at greater than 90% RH [13,31,44,52,56,84,116]. These opposing relationships for viability and RH may be owing to structural differences between virus types that were discussed previously. Some researchers have found no difference in infectivity across RHs, but these studies often involved unusual factors such as supplements to the media or experiments performed in the dark [14,16,17,23,25,29,43,49,53,67,81,117–120].

The relationship may depend on the composition of the suspending media, as virus stability has been shown to increase with the addition of mucins and proteins, removal of salts, or switching from culture medium to saliva in some studies [9,24,43,49,73]. Generally, influenza viruses have a U-shaped relationship between virus stability and RH, but the addition of proteins (fetal bovine serum (FBS), bovine serum albumin (BSA) or fetal calf serum (FCS)), mucins or HBE ECM can protect against decay (figure 1) [30,43,49]. However, the influence of these supplements can vary between virus strains and RHs (figure 1) [12]. Differences in decay kinetics across RHs for SARS-CoV-2 have been shown to depend on particle size, surface type and chemical composition of the suspension media [10,68,71,73]. Almost all of these studies used culture medium. As explored in §6, virus viability depends on droplet composition, and the results described above do not necessarily apply to physiologically relevant solutions. Additionally, the chemical composition of particles, along with RH, dictates their final physical state. Higher protein content increases the RH range over which aerosols and droplets exist in a semi-solid state [100,101]. The salt concentration and composition also affect transitions between states [86,121].

RH can affect other factors that potentially impact virus stability in aerosols and droplets. The first is the impact of RH on the final size of particles [11,87,122], although Merhi et al. [84] suggest saliva is a non-ideal mixture that results in a smaller increase in water fraction with RH. The equilibrium sizes would then be smaller and less dependent on RH than predicted with ideal mixture models [84]. Thus, while RH may have an impact on final particle sizes of ideal mixture solutions, more investigation with realistic respiratory fluids is needed.

RH also impacts the production and loss of chemical species that may influence virus viability. Researchers have observed the spontaneous formation of hydrogen peroxide (H_2_O_2_) on the surface of water droplets and shown that its concentration increases as RH increases [123]. RH can also impact ozone levels in the air, with higher observed initial ozone concentrations at low RH compared with high RH because the ozone interacts with water to form free radicals [50]. The impact of chemical species on virus viability will be further explored in §8.

RH and temperature play an important and multifaceted role in virus stability in aerosols and droplets. The effects of temperature are well understood. RH is an extrinsic factor that affects virus stability through its impact on evaporation and the final physical states and chemical compositions of particles. Many mechanistic details about changes in a virion’s microenvironment in an evaporating droplet and the effects on infectivity remain unknown.

### 5.3. Biphasic decay of virus

Numerous studies have reported a difference between the initial and longer term decay rates of viruses in particles, often corresponding to their ‘wet’ and ‘dry’ periods as they undergo evaporation [10,13,18,21,58,66,71,94,103,124–126]. In general, decay has been shown to be faster initially and then to slow, with the exception of a lower rate coefficient SARS-CoV-2 during the wet period (median half-lives of 5.76–42.08 h) compared with the dry period (median half-lives of 1.52–26.55 h) in one study [71]. This difference could be owing to the authors’ use of a correction factor to adjust for rapidly changing solute concentrations during the initial period. The difference in decay rates is more evident in aerosols, as evaporation occurs within minutes instead of hours, causing a large initial drop in viability followed by a slower period of decay [58].

In referring to this pattern as ‘biphasic decay,’ we use the term ‘phase’ to refer to the temporal evolution of virus decay in particles rather than the physical state of the particle. During the first phase, the droplet is liquid and undergoing evaporation; its physicochemical characteristics are dynamic. During the second phase, as previously mentioned, the particle may be liquid, semi-solid or solid, depending on RH. Physicochemically, it is at equilibrium, but virus decay may continue. Furthermore, a rapid loss in virus infectivity of approximately 50% has been observed at the time of efflorescence [10,21,65,66,74]. Thus, plots of virus infectivity as a function of time may appear to have a discontinuity between the first and second phases of decay if ambient RH is below the efflorescence RH. Haddrell *et al*. [74] described three phases of decay: a lag phase, a dynamic phase and a slow decay phase, where the dynamic phase corresponds to efflorescence or other rapidly changing conditions in a particle.

#### 5.3.1. Evaporating phase

During the evaporating phase, loss of water leads to increased concentrations of non-volatile solutes within the droplets that can lead to rapid virus inactivation [9,36,51,58,77,86,94,127–134]. However, Oswin *et al.* [10] observed no loss of infectivity of SARS-CoV-2 under increased concentrations of 10× Minimum Essential Medium (MEM) in bulk, suggesting that the increased concentration of solutes during evaporation does not account for the loss of infectivity in the aerosol phase. Salts have been implicated in virus decay [95], but Lin *et al*. [9] found an inconsistent effect of higher NaCl concentrations on enveloped and non-enveloped bacteriophages. Evaporation also increases the concentrations of organics, such as surfactants, mucin and protein, that can protect the virus [9,32,75,77,95,99,102,131,133]. Some have suggested that dehydration could inactivate the virus [28,29,86,92,132,135–138]. These results raise questions about mechanistic differences in decay between the evaporation and equilibrium phases.

The effect of evaporation may differ between aerosols and large droplets as well. Aerosols are smaller, evaporate more rapidly, and have a larger surface-area-to-volume ratio (table 2) compared with droplets deposited on a surface. This could account for the difference in decay rates between virions in aerosols and those in large droplets. Some researchers have suggested that virus aggregation at the air–particle interface drives inactivation [15,18,129,136]. Contact angle further affects droplets that are deposited on surfaces by affecting the surface area exposed to air and thus the evaporation rate [87,139,140]. RH and temperature have also been reported to affect evaporation, with high RHs and lower temperatures increasing the drying time and thereby favouring survival [8,87,92,105,106,139,141,142]. However, Merhi *et al*. [84] suggested RH may not have as large of an impact on evaporation and equilibrium droplet size over a range of 0–40 µm.

We calculated the first-order exponential decay constants (electronic supplementary material, tables S3–S5) for the studies shown in the network diagrams (figures 1 and 2, electronic supplementary material, figures S2 and S3). Most studies do not separate the decay phases, so the decay constants were calculated assuming a single decay rate. If decay is truly biphasic, then the estimated constants are an oversimplification. We are able to compare the effects of different factors on decay within a single study, but we are unable to compare values between studies. This is owing to a lack of standardization of methods and insufficient data provided about virus titre at time zero. While we split the influence of these factors into low, medium and high RH regimes, the decay constant can differ within a regime (e.g. 20% versus 30% RH).

#### 5.3.2. Equilibrium phase

Once evaporation ceases, the particles have reached equilibrium. During evaporation, materials may have accumulated around the peripheral ring of a large droplet on a surface in a process termed the ‘coffee ring effect’ [32,77,110]. Four stages of desiccation lead to the formation of four zones of a dried droplet: peripheral ring, zone of protein structures, protein gel and crystalline zone [32,77,100,110]. The desiccation and drying pattern of droplets may affect virus infectivity during evaporation [32].

The fate of viruses within particles during the equilibrium phase is not completely understood. The interactions between virions, inorganic compounds and organic compounds within droplets are complex, and researchers believe that they influence the location of viruses within a dried droplet and virus viability [32,77]. Some researchers observed an accumulation of virions around the periphery of the particle and suggest that the protein gel there provides a protective environment for the virus by reducing environmental exposure [32]. A limitation of these hypotheses is that they are often based on studies of viruses within cell culture media. Virus fate depends on the intermolecular forces on the virus within the droplet, so there is a need for studies with more realistic fluids and more realistic droplet sizes to provide a more accurate picture of virus inactivation in the equilibrium phase.

### 5.4. Summary of temperature, RH and evaporation effects on inactivation

Temperature has a clear inverse relationship with virus viability, while the relationship between RH and viability is more complex. In indoor environments relevant to transmission, temperature often falls within a narrow range, so RH will have a greater impact on viral stability. The observed relationships between RH and virus viability in particles depend on both the macrostructure and microstructure of the virion, and especially its microenvironment, which is strongly influenced by evaporation and the final physical state of the particle at equilibrium. RH impacts the duration of a particle’s liquid-like state that allows external factors, such as atmospheric gases, to partition into the particle, diffuse and interact with the virions. Then, RH further dictates the physical state of the particle once equilibrium is reached. The particle could exist in three different states, which affect other factors, such as chemical composition, pH and gas composition. These will be discussed in §§6–8. Most research into the effect of RH on virus viability and evaporation has been conducted in large droplets of culture medium. Again, moving to realistic respiratory fluids and smaller sized particles will help better define the significance of these relationships for transmission. The effect of RH on evaporation is often conducted on a smaller number of droplets. However, individuals exhale many droplets at once, and evaporation can be delayed owing to the high RH conditions within the turbulent gas cloud of a cough [7].

## 6. Chemical composition of the aerosol or droplet

Respiratory fluids can vary in composition from person to person and between infected and healthy individuals. In a simplified model, common components include salt, protein and surfactants [116]. However, real respiratory fluids are quite complex, and the chemical composition differs between specific fluids (e.g. saliva, bronchial mucus, nasal mucus and airway secretions) [143]. Often researchers use culture medium to conduct experiments on virus stability because these are the solutions commonly used to propagate virus stocks. Many have modified the concentrations of organics, such as proteins, mucins and surfactants, and inorganics, such as salts, in the media to investigate effects on virus stability.

### 6.1. Difference in media

Individual studies have tended to focus on the stability of one or more viruses at different environmental conditions in a single medium, and there has been limited research comparing virus stability in different suspending fluids. Some of the first such comparisons involved aerosolized FMDV suspended in cell culture medium, saliva, nasal fluid, milk and faecal slurry [111,112]. Other investigations have observed differences in stability when the virus is suspended in nasal secretions from an infected calf compared with nasal secretions from an uninfected calf [15], human saliva versus artificial saliva versus cell culture medium [96], and with the addition of HBE ECM [43,49]. Artificial saliva, unlike cell culture medium, contains carbohydrates that vitrify with decreasing RH and can provide further protection to virions [78]. However, artificial saliva lacks antivirals and other enzymes that are present in human saliva. On the other hand, some studies have found no difference in virus decay between suspension media [52,65,66,144]. Opposing results could be owing to the inclusion of small, potentially influential amounts of the original growth medium upon diluting a stock into different media.

#### 6.1.1. Inorganic salts

The effect of salt on virus stability depends on the type of virus and the humidity level. For enveloped viruses, some researchers attribute the ‘U-shaped’ relationship between viral viability and RH to salt concentrations. At low RH, salt is protective owing to efflorescence that inhibits inactivation [30,32,100,102,127]. At medium RH, salt has been observed to be detrimental to viruses, which some believe is owing to the increased concentration of salt during evaporation [24,30,32,54,86,127]. At high RH, salt appears not to be harmful to the virus, potentially owing to low solute concentrations [30,32,127]. For non-enveloped viruses, the harmful effect of salt appears to increase as RH decreases [9,29,145]. While some researchers hypothesize that salt concentration can explain the humidity relationship with viability, others have observed that changing the salt concentration has varying or no observable effect on decay in bulk suspensions, large droplets and aerosols [9,10,65]. In summary, salt concentration alone cannot explain viral decay. The effect of salt can also vary depending on the concentration of salt and the type of salt [9,31]. This includes varying stability with the addition of NaCl or KCl, which could be owing to the different efflorescence RHs between the two salts [31].

#### 6.1.2. Organic compounds

Proteins and other organic compounds make up the majority of non-volatile components of respiratory fluid [91]. Researchers have supplemented media with proteins in the form of FCS, BSA and FBS. Generally, proteins provide a protective effect for all types of viruses across all RHs [6,24,29,30,32,34,50,77,146]. Exceptions occurred at concentrations of protein below that present in saliva [54].

Mucin, a glycoprotein that has carbohydrates in addition to polypeptides, is secreted by epithelial cells in the airway and oral cavity. Mucin has been shown to provide a protective effect on virions depending on RH [30,34,75,112], and the effect may be independent of mucin concentration [75]. High organic content from mucin and proteins may protect viruses by promoting the formation of a semi-solid or glassy state at the air–particle interface that protects the virus from environmental factors [32,77,99,100,147,148]. This could be achieved by the formation of inclusions or highly porous microstructures around the virus [75,95,133]. Haddrell *et al*. [65] have proposed the opposite effect: crystallization of salts protects the virus while the remaining organic fraction reduces infectivity. While we still do not know the distribution of virions within aerosols and droplets, it is possible that virions could be in the outer, semi-solid shell where they are exposed to environmental factors that drive inactivation. The impact of solutes may also depend upon droplet size. Differences in evaporation rate as a function of size and solute concentration can lead to concentration gradients of widely varying magnitudes and lifetimes, and such gradients could impact virus stability [84].

Surfactants, which consist of phospholipids and amphiphilic proteins that are present in pulmonary fluid and saliva, have varying effects on stability between enveloped and non-enveloped viruses, depending on RH [9,50].

#### 6.1.3. Additional components

In limited studies that took place mostly in the 1970s and 1980s, researchers added other chemicals to suspension media, mainly to determine the importance of inactivation at the air–particle interface in aerosols and large droplets. Additives included peptone, phenylalanine, oxyethylene docosyl ether and oxyethylene octadecyl ether (OED), inositol, sucrose, glycerol, dimethyl sulfoxide (DMSO), methanol and polyethylene glycol [27,28,54,132,146,149]. Some chemicals, such as inositol and sucrose [54], had RH dependent effects on viability, but other chemicals, such as methanol and polyethylene glycol [132], had no effect. While these results provide some insight into mechanisms of virus inactivation, the additives are not naturally occurring.

### 6.2. Summary of particle composition effects on inactivation

Clearly, the suspending fluid composition has a considerable impact on virus stability through direct interactions between components of the fluid with a virion and their effect on the physical state of the particle. There is a lack of consensus on whether salts and organic compounds are protective or harmful, and interactions between specific compounds and RH are apparent. The use of cell culture medium can lead researchers to over- or under-estimate the stability of viruses and oversimplify the complex interactions within aerosols and large droplets although model fluids can be useful for identifying the role of specific compounds in virus inactivation. Future studies should employ a variety of respiratory fluids representing different regions of the respiratory tract. Differences in inactivation rates by fluid type may shed light on the potential for each to contribute to transmission.

## 7. pH

### 7.1. pH of droplets

The pH of droplets is not necessarily the same as in bulk solution because water at the air–water interface exhibits different structure and hydrogen bond dynamics relative to bulk water [150]. The large surface-area-to-volume ratio of droplets could affect their pH compared with bulk liquid. While the pH of bulk liquids is easily measured using pH strips or pH meters, the pH of aerosols and droplets has remained experimentally inaccessible until recently. Using gold nanoparticle-based pH probes and surface-enhanced Raman spectroscopy (SERS), Wei *et al*. [150] measured the spatial distribution of pH in droplets of phosphate buffer solution. The pH of the centroid was approximately 11, or 3.6 pH units higher than in the bulk solution [150]. The pH decreased from the centre towards the edge of the droplets, perhaps owing to the accumulation of protons at the air–water interface [150].

**Figure 4.**
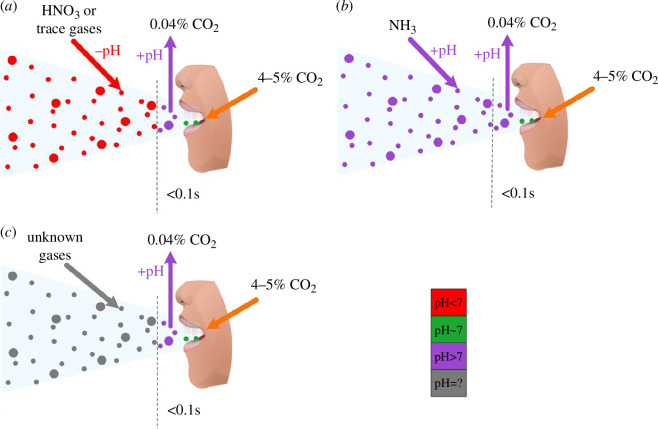
(*a*)–(*c*) Schematic of potential changes in pH in aerosols and droplets after exhalation into ambient air [10,55,65]. Figure created using ConceptDraw DIAGRAM, by CS Odessa.

Two hypotheses have emerged in the literature about the pH of respiratory aerosols and droplets (figure 4). They begin with the fact that aerosols and droplets experience a rapid change in gaseous carbon dioxide (CO_2_) concentration when they are exhaled from the respiratory tract into ambient air, transitioning from a CO_2_ mixing ratio of approximately 4–5% CO_2_ to approximately 0.04% CO_2_. Oswin *et al*. [10] hypothesized that the drop in gas-phase CO_2_ concentration causes partitioning of dissolved CO_2_ into the gas phase and a shift in the bicarbonate system that leads to a rise in pH. Oswin *et al.* [10] also demonstrated that when bulk culture medium is exposed to open air in thin films or has air bubbled through it, the pH increases. The pH of aerosolized saliva is expected to increase dramatically to 11 upon expulsion from the respiratory tract [10]. Luo *et al*. [55] hypothesized that the pH rises only for a very short time (less than 0.3 s) before trace gases in indoor air, such as nitric acid (HNO_3_), interact with the droplets, causing the droplets to become more acidic [55]. In addition, David *et al*. [151] modelled the acidification of 0.4, 2 and 10 µm aerosols upon exhalation and showed the time to reach pH 4 increased with increasing droplet size. Neither of these hypotheses has been experimentally proven because we are not yet capable of measuring pH in droplets with high temporal resolution. Laboratory studies do not usually simulate the transition from 5% CO_2_ to 0.04% CO_2_, so alkalization that occurs in real systems probably is not being captured. Furthermore, these studies have not considered the role of phosphate, which is present in saliva at a concentration of 5.1 mM [152] and may buffer changes in pH. The predominant buffering system in saliva is dependent on the type of saliva: stimulated (produced from eating and chewing) versus unstimulated (resting and spitting) [153]. The bicarbonate system is the most important system for stimulated saliva while the phosphate system is important for unstimulated saliva, which is approximately 2/3 of the total saliva in our mouths [154]. We recommend pursuing this line of questioning further, as pH is potentially a driving factor in virus inactivation.

### 7.2. pH impact on virus stability

There is ample evidence that pH is a driver of inactivation of viruses in aerosols and droplets. Acidic conditions can lead to the inactivation of enveloped viruses, where the extent of inactivation depends on the specific virus (IAV versus SARS-CoV-2 versus HCoV versus Phi6) (figures 1*c* and 2, electronic supplementary material S3*a*) [9,55]. However, the non-enveloped MS2 phage remained stable across all pHs and RHs (electronic supplementary material, figure S2*a–c*) [9]. This difference may be owing to the different mechanisms of virus entry into host cells. During endosomal entry, influenza virus must go through an acid-induced conformational change of the HA protein that could be induced early by a pH change in the droplet, but coronaviruses can achieve fusion either by a pH-driven conformational change or through cleavage by host proteases [55]. The HA activation pH threshold varies between human, swine and avian influenza viruses [46]. David *et al*. [151] analysed three different viral proteins, as well as lipids and genomes, to assess the viral integrity of IAV under acidic pH conditions [151]. The viral proteins rather than the lipids and genomes experienced measurable changes associated with pH-mediated decay. Interestingly, they proposed two-step kinetics with an initial sharp drop in infectivity in less than 10 s associated with the irreversible HA conformation followed by a slower decay of over 30 s caused by changes to M1 and associated capsid disassembly [151]. David *et al*. [151] further showed that the pH-induced conformational changes may not lead to inactivation of all viruses, depending on whether the change to the protein involved is reversible or not.

Luo *et al*. [55] suggested that acidic conditions in droplets drive virus inactivation, but Oswin *et al*. [10], Haddrell *et al*. [65] and Alexander *et al*. [75] have demonstrated the effect of alkaline pH on SARS-CoV-2 and MHV inactivation. Oswin *et al*. [10] observed that SARS-CoV-2 in bulk suspensions remained stable at pH 9 but decayed similarly at pH 11 to virus in levitated droplets at 90% RH. Haddrell *et al*. [65] demonstrated that the introduction of HNO_3_ to the system had minimal effects on virus stability, contrary to the prediction of Luo *et al*. [55]. Haddrell *et al*. [65] further demonstrated that increasing the gaseous CO_2_ concentration increased the stability of SARS-CoV-2 in aerosols [74]. Results align with a study on SARS-CoV-1 that observed infectivity was sensitive to both acidic conditions (pH 1–3) and alkaline conditions (pH 12–14) but stable at pH 5–9 [63]. Further investigations have revealed that both influenza virus and SARS-CoV-2 remain infectious across wide ranges of pH (6.8–9.1 and 3–10, respectively) [54,69], while FMDV experiences accelerated decay under alkaline pH conditions [111,125].

### 7.3. Summary of pH effects on inactivation

Clearly, extreme pH can cause virus inactivation. The pH of respiratory droplets appears to rapidly increase upon transitioning from the respiratory tract to ambient conditions, but the magnitude and dynamics of the change have not been directly measured. Methods to measure a rapid change in pH in droplets of physiologically relevant size and composition do not currently exist. Once we are able to characterize the dynamics of pH in droplets, we can build a more thorough mechanistic understanding of the impact of pH on virus inactivation. The impact of pH on stability is more important, probably, while a particle is still wet. Once again, we see that RH is closely tied to a factor of inactivation. Thus, the effect of pH on virus viability could be RH dependent. Many of the recent studies on pH have been conducted at high RH with liquid particles. Investigation is needed into the role of pH in semi-solid particles at medium RH. At low RH, pH may have less of an effect because particles evaporate quickly, and the solid state of the particle will limit the diffusion of gases in and out of the particle.

## 8. Atmospheric composition

The air in the Earth’s atmosphere is approximately 78% nitrogen and 21% oxygen. Many other trace gases, such as organic acids, reactive oxygen species (ROS) and CO_2_, are also present. The effect of water vapour is discussed in §5.2. Aerosols and droplets interact with the surrounding air, but there is limited research into how the composition of the air could affect virus viability, aside from studies on intentional disinfection with high levels of vaporized hydrogen peroxide or other compounds.

### 8.1. Gas-phase composition

Acidic and basic gases may partition into droplets and affect their pH. Luo *et al*. [55] modelled the evolution of physiochemical conditions, including organics, nitrate (NO_3_^-^), water (H_2_O), total ammonium (NH_4_^+^) and chloride (Cl^−^), within a respiratory particle and how these would affect its acidity and the inactivation of IAV and SARS-CoV-2. Inactivation of both viruses increased with the addition of 50 ppb of HNO_3_ in the gaseous phase [55]. Haddrell *et al*. [65] experimentally exposed SARS-CoV-2 in levitated droplets to HNO_3_ over 40 min and observed a minimal effect on stability. Ammonia gas, which would raise the pH of droplets, did not affect avian influenza virus decay [44].

Naturally occurring ROS, such as peroxides, superoxide, hydroxyl radical, singlet oxygen and alpha oxygen, can damage DNA, RNA and proteins [155], and may play a role in virus inactivation [129,156]. Potential sources in the air include species generated by photolysis and other reactions. Studies have examined the effect of simulated sunlight on the inactivation of viruses in aerosols [53,66,67,72]. There was little to no decay in darkness for SARS-CoV-2 and influenza virus across temperatures and RHs tested, whereas decay increased with increased light levels [53,66,67,72]. This raises the potential for ROS generated by sunlight to be a driving factor in virus inactivation.

Aqueous ROS that have been observed to arise from interactions at the air–water interface of pure water droplets could also be a factor. Recent work suggests that H_2_O_2_ forms spontaneously on the surface of water microdroplets [123,157,158]. A spray of small water droplets acts as a bactericide, possibly owing to the ROS [159]. These results raise the possibility of aqueous ROS driving inactivation of viruses. H_2_O_2_ in the aqueous phase and ozone in the gaseous phase each have an observable relationship with RH that is probably owing to the availability of water to react with chemicals in the air [50,123,160,161]. Dulay *et al*. [123] observed higher ROS concentrations in microdroplets at medium RH, and Davidse & Zare [129] suggested that ROS can explain the U-shaped relationship between RH and virus viability. Turgeon *et al*. [162] exposed aerosolized phages to nebulized 3% H_2_O_2_ and observed little impact on phage infectivity. This suggests that aqueous ROS may have a larger impact than gaseous ROS, but further investigation is needed.

Researchers have observed no differences in virus inactivation between nitrogen gas and ambient air atmospheres [24,28,65,163]. If aqueous ROS were a driver of inactivation, we would expect to see differences between the two atmospheres because we expect much lower concentrations of ROS in nitrogen than in ambient air. However, Mehrgardi *et al*. [158] have shown that H_2_O_2_ forms on the surface of water microdroplets in a nitrogen atmosphere, although in lower concentrations. The duration of these experiments was short, ranging from 5 s to 1 h; ROS could play a more significant role in long-term inactivation. We recommend further investigation into the role of gaseous and aqueous ROS in the inactivation of viruses in aerosols and droplets.

Barlow [132] investigated the effect of DMSO, an ROS quencher, on the stability of FMDV in aerosols and observed recovery at RH less than 45%, where FMDV decays most rapidly. Much remains unknown about the potential role of ROS in virus inactivation, and the potential interactions between ROS and virions in aerosols and droplets are very complex.

### 8.2. Particle-phase composition

In an investigation of the effect of fine particulate matter (PM_2.5_) on viruses, Groulx *et al*. [37] observed decreased infectivity of the enveloped bacteriophage Phi6 compared with the non-enveloped bacteriophage PhiX174. This again demonstrates the difference in stability between enveloped and non-enveloped viruses.

### 8.3. Summary of gaseous composition effects on inactivation

The effects of gas- and particle-phase composition of the surrounding atmosphere have not been studied extensively. Exposure to sunlight accelerates virus inactivation; radiation could directly affect a virion, or it could produce photochemical products that lead to inactivation. The differing results for HNO_3_ between modelling and experimental emphasize the need for further investigation into this aspect. While the hypothesis of spontaneous ROS formation on the surface of droplets is captivating, researchers must provide evidence that this phenomenon occurs in respiratory particles. Air is chemically complex and varies depending on location, making it difficult to reach definitive conclusions about this mechanism. Much more research is needed into the effects of atmospheric composition on virus viability.

## 9. Conclusion: summary and knowledge gaps

Despite the large number of studies on virus stability in aerosols and large droplets, a mechanistic understanding of virus inactivation remains elusive. Clearly, inactivation is accelerated at higher temperatures and slowed at lower temperatures [35,69,71]. Other environmental conditions, such as RH and gaseous composition, also impact virus stability in ways that are not as clearly defined, while the composition of the suspending liquid is a critical factor [9,22,37,43,55,96,112]. Extreme pH conditions, acidic and alkaline, lead to virus inactivation [55,63,79]. Droplet size also plays a role in viral persistence and the kinetics of decay [21].

We provide the following recommendations for advancing understanding of this field. The stability of viruses should be tested in natural respiratory fluids like saliva, nasal fluid and airway surface liquid. Fluid composition has an important impact on viral decay and must be considered when drawing conclusions about viral persistence. Stability of viruses should be tested in physiologically relevant particle sizes (less than 5 μm representing small particles, 5–100 μm representing large particles, and 100–1000 μm representing large droplets). Size dictates where the particles will deposit in the respiratory tract, which impacts pathogenesis. Extrapolation of results involving surrogate viruses or laboratory-adapted strains of viruses to pathogens should be limited, although these results may provide further insight into broader principles of virus inactivation. A broader array of viruses beyond SARS-CoV-2 and influenza virus merits study. Many other viruses are significant for public health (e.g. rhinovirus and RSV), and much less is known about their airborne stability. Dynamic changes in pH in aerosols and large droplets should be directly probed. The location of virions in aerosols and droplets should be investigated because their composition may be spatially heterogeneous, and virus inactivation depends on the local microenvironment. The role of aqueous and gaseous ROS in virus inactivation should be investigated further.

New approaches have enabled considerable advances in understanding virus inactivation in aerosols and droplets in recent years, but we should maintain caution about oversimplifying the system. Rather than a single factor driving inactivation, it is likely that a combination of interacting factors affects virus stability. Furthermore, the widely varying time scales of different processes may alter their relevance for short-range airborne transmission, where rapid evaporation and efflorescence may drive initial inactivation, and long-range airborne or fomite transmission, where RH may play a more important role. The impact of environmental factors can differ between viruses, which will in turn affect both the aerosol size contributing to transmission as well as the viral load being delivered. Once we comprehend the mechanisms of inactivation across a broader array of viruses within various environmental contexts and across biological droplet matrices, we can begin the next step: introducing interventions designed to enhance the inactivation of specific viruses in the environment. Non-pharmaceutical interventions will remain crucial for slowing the transmission of new viral threats while vaccines are being developed.

## Data Availability

All data supporting this article are from published articles in the review and are included in tables in the supplementary material [164].
